# A Peculiar Case of Recurrent Coronary Artery Thrombosis

**DOI:** 10.7759/cureus.29357

**Published:** 2022-09-20

**Authors:** Samuel Nwaobi, Zachary Wood, Aarushi Kalra, Sophia Nguyen

**Affiliations:** 1 Family Medicine, Piedmont Columbus Regional Midtown, Columbus, USA; 2 Family Medicine, Edward Via College of Osteopathic Medicine, Auburn, USA; 3 Internal Medicine, Philadelphia College of Osteopathic Medicine, Suwanee, USA

**Keywords:** drug eluting stent, dual anti-platelet therapy, st-elevation myocardial infarction (stemi), genetics of coronary artery disease, coronary artery thrombosis, heterozygous factor v leiden mutation

## Abstract

Coronary artery thrombosis is a phenomenon physicians have studied throughout the years. The different risk factors that play a role in the formation of an atherosclerotic plaque leading to coronary artery blockage are vast and can affect the patient significantly if not examined and diagnosed carefully. The objective of this case report is to highlight this unusual occurrence of repeated coronary artery thrombosis. A 54-year-old Caucasian female presented to the emergency department with a one-day history of sharp chest pain in the substernal area that radiated between her shoulder blades and left arm. Despite being on dual antiplatelet therapy, an electrocardiogram (ECG) showed an inferior ST-elevation myocardial infarction (STEMI). Her medical history was extensive with factor V Leiden deficiency, hyperhomocysteinemia, recurrent deep vein thrombosis (DVT), and a family history of myocardial infarction. The patient was taken to the cardiac catheterization lab based on these characteristics. The patient was diagnosed with a 100% thrombosis in the distal right coronary artery (RCA), which was stented nine months before this current presentation. The patient had been compliant with all her medications from her previous stent placement. A new drug-eluting stent (DES) was inserted, and the patient was placed on prasugrel and apixaban. This was a very interesting topic for a case report due to the time frame of repeat thrombosis in a coronary artery with a DES and the patient’s underlying hypercoagulable state. There are few cases of same vessel restenosis post-DES placement. Our case highlights the need for further research into the prevalence of genetic risk factors in coronary artery thrombosis and the need to investigate the efficacy of different anticoagulation therapies in patients with factor V Leiden thrombophilia.

## Introduction

Stent thrombosis (ST) is an adverse clinical incident that often results in ST-elevation myocardial infarction, and mortality rates are estimated to be as high as 20% to 40%. One of the most significant advances in the development of stent therapy was the evidence that showed dual antiplatelet therapy (DAPT) with aspirin and an adenosine diphosphate (ADP)-receptor inhibitor could decrease stent thrombosis and bleeding complications [1,2]. Rates of early (within one month) and late (up to 12 months) ST have been cut by half in recent years from 3% to 1.5% [3]. For early ST, procedural risk factors are the most critical. Patient-specific risk factors, such as left ventricular function, diabetes mellitus, and compromised response to ADP-antagonist treatment [4], are essential for late ST. Coagulation homeostasis is a constant balance between pro and anticoagulant behaviors. Thus, medical conditions and gene anomalies could tilt the balance in support of thrombosis in any abnormal coagulation state or if there is a lack of appropriate antithrombotic treatment. The latest recommendations are dual antiplatelet therapy for 12 months after acute coronary syndrome and extended treatment in patients with a low risk for bleeding [5,6]. Patients treated with up-to-date antiplatelet drugs, peri-procedural antithrombin regimens, and contemporary drug-eluting stents (DES) typically have exceptional results over the short to medium term. 

Factor V Leiden is an inherited condition due to a gene mutation of the F5 gene that increases the risk of abnormal clotting of blood. This thrombophilia is more associated with developing venous clots compared to arterial clots. Factor V plays a crucial role in the coagulation cascade system through its interaction with activated protein C (aPC). Factor V Leiden can be inherited either through a homozygous or heterozygous pattern. There is a 10% to 15% prevalence in people with European ancestry and less in Native Americans and African Americans. This thrombophilia is usually diagnosed through an aPC resistance test or coagulation screening.

Along with rare coagulation factor deficiencies, hyperhomocysteinemia is another rare occurrence in the patient population. Hyperhomocysteinemia is correlated to vitamin deficiencies such as vitamins B12 and B6, and folate deficiency. This can inevitably lead to heart disease, stroke, and possibly dementia. A blood test for homocysteine and vitamin panel should be collected to diagnose this condition. Factor V Leiden mutation and hyperhomocysteinemia are considered independent risk factors that may lead to harmful complications involving the cardiovascular system and neurological problems. Our case report highlights the risk of repeated coronary artery thrombosis despite DAPT in a patient with a medical history of factor V Leiden and hyperhomocysteinemia who had a drug-eluting stent placed in the same coronary vessel. 

## Case presentation

A 54-year-old Caucasian female presented to the emergency department with a chief complaint of sharp chest pain in the substernal area that radiated between her shoulder blades and left arm. She mentioned that she started having headaches the night before, but on waking up, she began to experience chest pain with associated diaphoresis and shortness of breath. She promptly got driven to the hospital by her family. The patient provided significant history regarding her chronic medical conditions and family and social history. The patient mentioned having coronary artery blockages found incidentally during cardiac clearance for shoulder surgery nine months prior. She subsequently underwent coronary angioplasty of the occluded right coronary artery (RCA) with the insertion of a drug-eluting stent, and the patient was discharged home on aspirin, clopidogrel, and apixaban. The patient’s other chronic illnesses included hyperhomocysteinemia, factor V Leiden deficiency, and multiple incidents of DVTs within three and four years of each other, with the last occurrence reported in December 2019. She was also diagnosed with obstructive sleep apnea, hypertension, and hyperlipidemia. Her previous surgery and hospitalizations include a hernia repair, thrombectomies for her DVTs, skin graft, neurostimulator implantation, rotator cuff repair, and coronary artery stent placements. The patient was a current smoker and had a 54-pack-per-year smoking history. She does not consume alcohol. The patient’s father had a stroke and died at 43 from his third myocardial infarction. Her father’s sister and brother had myocardial infarctions before they were 50. 

**Figure 1:**
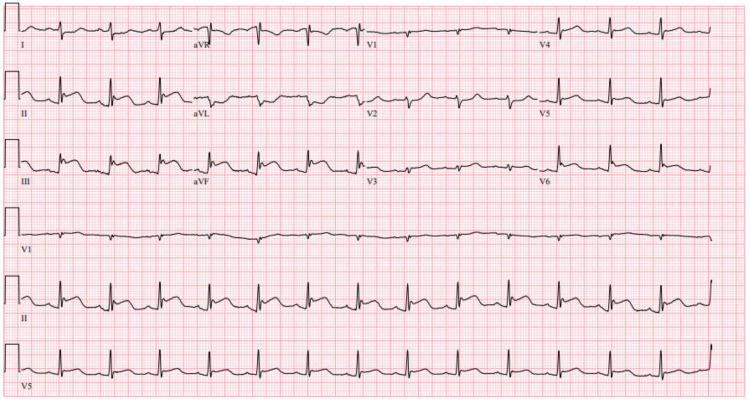
ECG showing ST elevation in Leads II, III, aVF ECG: Electrocardiogram, aVF: Augmented vector foot

Upon admission to the emergency department, the patient appeared to be in acute distress, and her electrocardiogram (ECG) showed ST elevation in leads II, III, and augmented vector foot (aVF) (Figure 1).

The patient’s vitals showed blood pressure 225/103 mmHg, heart rate of 90 beats per minute, respiratory rate of 31 breaths per minute, and oxygen saturation of 96% on room air. Her complete blood count (CBC), comprehensive metabolic panel (CMP), international normalized ratio (INR), hemoglobin A1C, and bilirubin levels were within normal limits. Initial high sensitivity troponin was noted to be significantly elevated and progressively worsened (Table 1).

**Table 1:** Markedly elevated troponin levels

Time	Troponin levels (ng/L)
0 hour	1,692
2 hours	72,292
6 hours	94,599

**Figure 2:**
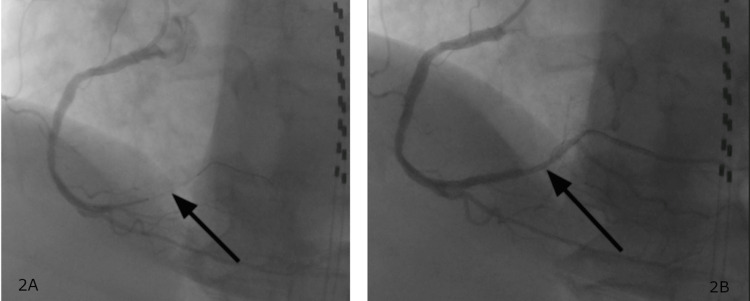
Image 2A shows the distal RCA 100% occluded on coronary artery angiogram. Image 2B features the coronary artery angiogram that shows improved blood flow through the coronary artery vessels post RCA angioplasty. RCA: Right coronary artery

The medications given in the emergency department included aspirin 325mg, morphine 4mg intravenous (IV), ondansetron 4mg IV, nitroglycerin 0.4mg, and heparin 4,000U IV. The patient required a nicardipine drip due to a hypertensive emergency. The patient was transferred to the cardiac catheterization laboratory for coronary angiography and percutaneous coronary intervention. Her cardiac catheterization results showed an ostial left anterior descending (LAD) lesion 35% stenosed, distal LAD lesion 40% stenosed, mid circumflex lesion 25% stenosed, proximal RCA lesion 15% stenosed, mid-RCA lesion 35% stenosed, and distal RCA lesion 100% stenosed (Figure 2, A). The lesion was a type-A thrombotic lesion located at a bifurcation with a side branch. Acute in-stent thrombosis was noted, and stenosis was measured by visual reading. Intravascular ultrasound (IVUS) or optical coherence tomography (OCT) was not performed on the lesion. The guidewire crossed the lesion, and pre-stent angioplasty was performed. There was no pre-intervention antegrade distal flow (thrombolysis in myocardial infarction (TIMI) grade 0). A new drug-eluting stent was placed in the RCA with improved post-angioplasty blood flow (TIMI Grade 3) with 0% residual stenosis post-intervention (Figure 2, B). Left ventricular end-diastolic pressure was severely elevated at 41mmHg. The patient’s echocardiogram showed a left ventricular ejection fraction of 45% to 50%. Left ventricular (LV) systolic function was mildly decreased along with mild concentric LV hypertrophy and grade I diastolic dysfunction. 

During the patient’s stay, she was observed overnight in the medical intensive care unit for recovery and was transferred to the medical unit the next day. On reevaluation, she denied any chest pain or shortness of breath. Cardiology recommended discharging the patient on apixaban 5mg twice daily and prasugrel 10mg once daily. The patient was discharged with adequate follow-up with her primary care physician and cardiologist.

## Discussion

Factor V Leiden is the most common cause of inherited thrombophilia [7]. Factor V Leiden mutation is a missense substitution that causes a replacement of arg506 (CGA) by gln (CAA). This substitution removes one of the aPC cleavage sites on the heavy chain of factor V. Consequently, factor V Leiden is now in an atypical form, making it resistant to the function of aPC, and is unable to halt factor V from producing more fibrin. Therefore, when the coagulation cascade is started in individuals with mutated factor V Leiden, it is inactivated slower than in individuals with a typical form of factor V, producing a heightened risk of thrombosis. The mutation's heterozygosity (GA genotype) is seen in approximately 5% of the general population, while the homozygosity (AA genotype) is seen in less than one percent. Our patient most likely had the heterozygous mutation due to her father’s history of repeated myocardial infarctions at a young age. Factor V Leiden is an established risk factor for venous thromboembolism [8]. Still, its role in arterial thrombosis [9] is controversial, and limited data is available on its role in the occurrence of major adverse cardiac events after coronary stenting, which makes this an interesting case. Factor V Leiden has been shown to increase the risk for myocardial infarction in a population-based case-control study in young women aged 18 to 44 with a smoking history [10]. The theory this paper proposed was that estrogens additionally reduce the inactivation rate of factor V by aPC, causing increased resistance to aPC. 

Platelet function and resistance to antiplatelet drugs are additional critical aspects in arterial thrombotic events [11]. Patient response to clopidogrel has recently garnered attention, with an impressive correlation between high on-clopidogrel platelet reactivity and ST in the face of strict compliance to DAPT [12]. Clopidogrel is a prodrug that needs metabolism to form its active thiol product. In a study on the effectiveness of clopidogrel in patients with a history of thrombosis, it was suggested that switching to prasugrel, ticagrelor, or higher-dose clopidogrel may be the ideal option. “In these special patients, we believe that the benefit of more potent antiplatelet therapy outweighs the increased risk of bleeding in most patients.” [13]. Current guidelines suggest DAPT, aspirin, and one ADP-receptor antagonist (i.e., clopidogrel, prasugrel, or ticagrelor), for antiplatelet therapy [14]. Initially, the patient was on warfarin for 30+ years but was switched to clopidogrel and aspirin after her first set of stent placements. The Trial to Assess Improvement in Therapeutic Outcomes by Optimizing Platelet Inhibition with Prasugrel-Thrombolysis in Myocardial Infarction (TRITON-TIMI) 38 study indicated that ST was more frequent on clopidogrel compared to prasugrel (142/6653 vs. 68/6745), which could be a factor as to why our patient had a thrombus form after her change in medications [15]. Regarding the myocardial infarction the patient experienced, research suggests that the 30-day mortality following ST is high (10% to 25%). It has been shown that almost 20% of patients with ST have a repeated event [16]. The primary explanation for poor responsiveness to clopidogrel is deficient clopidogrel active thiol metabolite production due to cytochrome P450 2C19 (CYP2C19) gene variation [12]. The CYP2C19 genetic polymorphisms are associated with decreased clopidogrel responsiveness and negative clinical consequences [17-19]. Studies have shown that if ST occurs on dual antiplatelet therapy that includes clopidogrel, substituting with a more potent drug such as prasugrel or ticagrelor is an equitable alternative [20]. In this case, the patient was discharged on prasugrel after experiencing the repeat ST on dual antiplatelet therapy with clopidogrel, and she was continued on apixaban, considering her hypercoagulable state.

## Conclusions

Recurrent coronary artery stent thrombosis within months of placement is not common. Our patient’s case highlights the need for close follow-up and proper anticoagulation in patients with thrombophilia conditions, such as factor V Leiden. Thrombophilia screening and genotype-guided anticoagulation to decrease the risk of stent thrombosis are not currently standard practices among physicians. However, they might be considered based on an individualized patient risk profile to prevent stent thrombosis. Further studies of this acute phenomenon in this special population could provide more information on analyzing the different risk factors and diseases associated with thrombosis of a previously placed drug-eluting stent while on dual antiplatelet therapy.
